# Combined assessment of carotid intima–media thickness and epicardial fat thickness for cardiovascular risk stratification in asymptomatic South Indian adults

**DOI:** 10.3389/fcvm.2026.1841043

**Published:** 2026-09-03

**Authors:** Ramesh Sankaran, Dolly Agarwal, Rahul Mittal, Shankar Srinivasan, Nagendra Boopathy Senguttuvan, Alex Karev, Sadhanandham Shanmugasundaram, Balasubramaniyan Jayanthi Venkata, Vinod Kumar Balakrishnan, Preetam Krishnamurthy, Sadagopan Thanikachalam, Murthy Jayanthi Satyanarayana

**Affiliations:** 1 Cardiac Care Centre, Sri Ramachandra Institute of Higher Education and Research, Chennai, Tamil Nadu, India; 2 Department of Health Informatics, Rutgers School of Health Professions, Rutgers Health, Rutgers University, Piscataway, NJ, United States; 3 Division of Cardiology, Robert Wood Johnson Medical School, Rutgers University, New Brunswick, NJ, United States

**Keywords:** cardiovascular imaging, cardiovascular risk factors, carotid intima-media thickness, epicardial fat thickness, South Asian population, vascular remodelling

## Abstract

**Background:**

Carotid intima-media thickness (cIMT) and epicardial fat thickness (EFT) are non-invasive imaging markers associated with cardiovascular risk. This study evaluated the associations between cIMT, EFT, individual cardiovascular risk factors, cumulative cardiovascular risk-factor burden in asymptomatic adults undergoing routine cardiovascular health assessment.

**Methods:**

A cross-sectional study was conducted in 925 asymptomatic adults aged ≥18 years undergoing routine cardiovascular health evaluations. Participants were classified into a control group without cardiovascular risk factors (*n* = 179) and a study group with one or more cardiovascular risk factors (*n* = 746). Carotid intima-media thickness was measured using carotid ultrasonography, and epicardial fat thickness was assessed by transthoracic echocardiography. Associations between imaging measurements, cardiovascular risk factors, cumulative cardiovascular risk-factor burden, were evaluated using descriptive and comparative statistical analyses.

**Results:**

Participants with one or more cardiovascular risk factors demonstrated significantly greater right cIMT (0.681 ± 0.186 mm vs. 0.576 ± 0.144 mm, *p* < 0.001), left cIMT (0.711 ± 0.193 mm vs. 0.587 ± 0.143 mm, *p* < 0.001), and EFT (5.45 ± 2.30 mm vs. 4.24 ± 1.85 mm, *p* < 0.001) than controls. Hypertension, diabetes mellitus, and dyslipidaemia were each significantly associated with increased cIMT and EFT (all *p* < 0.001). The prevalence of abnormal cIMT increased from 2.2% in participants younger than 40 years to 28.0% in those older than 60 years (*p* < 0.001). Progressively greater cIMT and EFT were observed with increasing cardiovascular risk-factor burden (both *p* < 0.001). Mean left cIMT was modestly but significantly greater than mean right cIMT (mean paired difference 0.026 mm, *p* < 0.001).

**Conclusions:**

Asymptomatic adults with cardiovascular risk factors demonstrated significantly greater carotid intima-media thickness and epicardial fat thickness than those without risk factors. Increasing age and cumulative cardiovascular risk-factor burden were associated with progressive increases in both imaging markers. These findings support the use of cIMT and EFT as complementary non-invasive imaging markers that may improve cardiovascular risk stratification alongside conventional risk assessment.

## 1 Introduction

Cardiovascular disease (CVD) remains the leading cause of mortality worldwide, accounting for approximately 19.8 million deaths annually and posing a substantial burden in low- and middle-income countries, including South Asia (1–3). Atherosclerosis is the principal pathological process underlying most cases of ischaemic heart disease, cerebrovascular disease, and peripheral arterial disease, and frequently progresses silently for many years before the onset of clinical symptoms (4). South Asian populations experience a disproportionately high burden of premature atherosclerotic cardiovascular disease (ASCVD), with approximately a two-fold higher risk of ASCVD than individuals of European ancestry, earlier disease onset, and a greater prevalence of cardiometabolic risk factors, including hypertension, diabetes mellitus, central adiposity, and physical inactivity (3, 5–8). Consequently, conventional cardiovascular risk prediction models developed predominantly in Western populations may underestimate cardiovascular risk in South Asians, highlighting the need for improved approaches to identify subclinical atherosclerosis before the development of overt cardiovascular disease (5, 6).

Atherosclerosis develops progressively over many years and frequently remains clinically silent until the occurrence of major adverse cardiovascular events such as myocardial infarction or stroke (4). Early identification of subclinical vascular disease is therefore essential to facilitate timely preventive intervention. Although conventional cardiovascular risk prediction tools, including the Framingham Risk Score (FRS) and Systematic Coronary Risk Evaluation (SCORE2), provide valuable estimates of cardiovascular risk, they may not adequately identify individuals with early subclinical disease, particularly among South Asian populations in whom current risk prediction models may underestimate cardiovascular risk (5, 9, 10). Consequently, non-invasive vascular imaging has become increasingly important for refining cardiovascular risk assessment beyond traditional risk factors. Among these modalities, carotid intima-media thickness (cIMT), measured using high-resolution B-mode ultrasonography, is a well-established, non-invasive and reproducible marker of subclinical vascular disease that has consistently been associated with future cardiovascular events independent of traditional cardiovascular risk factors (11–15). Although cIMT does not directly assess coronary atherosclerosis, it is widely recognised as a surrogate marker of systemic atherosclerotic burden and provides incremental value for cardiovascular risk stratification (11–16).

Accumulating evidence has established epicardial fat thickness (EFT) as an important non-invasive imaging marker associated with cardiometabolic risk and coronary artery disease (17–22). Epicardial adipose tissue is a metabolically active visceral fat depot located between the myocardium and the visceral pericardium that contributes to local inflammatory and atherosclerotic processes through the secretion of adipokines and pro-inflammatory cytokines (18, 20, 23). Increased EFT has been associated with obesity, insulin resistance, metabolic syndrome, coronary artery disease, and systemic inflammation (17–22). Recent prospective studies together with a contemporary systematic review and published meta-analysis have demonstrated that increased epicardial adipose tissue is associated with coronary artery disease severity and myocardial ischaemia, further supporting its role as a clinically relevant imaging marker (17–19, 21–23). Published studies have demonstrated a positive association between EFT and cIMT, suggesting that these complementary imaging markers reflect shared mechanisms of subclinical atherosclerosis and cardiometabolic risk (24, 25). Although EFT can be quantified using computed tomography (CT) or magnetic resonance imaging (MRI), transthoracic echocardiography provides a practical, cost-effective, and radiation-free alternative for routine clinical assessment, with recent prospective studies further supporting its clinical applicability (18, 20).

Despite growing interest in cIMT and EFT as complementary non-invasive imaging markers of subclinical atherosclerosis and cardiovascular risk (26), relatively few studies have evaluated their combined utility in asymptomatic populations, particularly in South Asia, where cardiovascular disease develops at a younger age and often follows a more aggressive course (27).

To our knowledge, this study provides one of the most comprehensive evaluations of carotid intima-media thickness (cIMT), epicardial fat thickness (EFT), and cumulative cardiovascular risk-factor burden in asymptomatic adults from South India undergoing routine cardiovascular risk stratification. The objectives of this study were to: (1) evaluate cIMT and EFT as complementary non-invasive imaging markers associated with cardiovascular risk burden and vascular remodelling. (2) examine the associations between these imaging markers and established cardiovascular risk factors; (3) evaluate the relationship between cumulative cardiovascular risk-factor burden and changes in cIMT and EFT; and (4) compare left and right cIMT measurements within the study population.

## 2 Materials and methods

### 2.1 Study design and population

This cross-sectional observational study was conducted between December 2023 and May 2024 at the Department of Cardiology, Sri Ramachandra Institute of Higher Education and Research, Chennai, India. Consecutive adults attending routine cardiovascular health assessments were prospectively recruited. Asymptomatic status was defined as the absence of known cardiovascular disease and the absence of symptoms suggestive of myocardial ischaemia, heart failure, cerebrovascular disease, or peripheral arterial disease at the time of recruitment.

Established cardiovascular risk factors were assessed using definitions consistent with the INTERHEART study framework (6). All participants underwent a standardized cardiovascular evaluation including transthoracic 2D echocardiography and carotid Doppler ultrasonography using a Vivid E95 ultrasound system (GE Healthcare). EFT was measured by transthoracic echocardiography, whereas cIMT was assessed using carotid ultrasonography.

Participants were classified according to cardiovascular risk-factor status. Individuals without identified cardiovascular risk factors were assigned to the control group, whereas those with one or more cardiovascular risk factors comprised the study group. The cardiovascular risk-group classification, participant eligibility criteria, and baseline clinical assessments are summarised in Table 1.

**Table 1:** Study group classification, participant eligibility, and clinical assessment.

Category	Details
Cardiovascular risk groups	Control group: Participants with no cardiovascular risk factors.
	Study group: Participants with one or more cardiovascular risk factors.
Inclusion criteria	Adults ≥18 years attending routine cardiovascular health assessment; asymptomatic at the time of evaluation; able to provide informed consent.
Exclusion criteria	Age <18 years; inability to provide informed consent; known coronary artery disease; heart failure; cerebrovascular disease; peripheral arterial disease; significant valvular heart disease; chronic kidney disease.
Clinical assessment	Standardized medical history, physical examination, medication history, smoking status, and family history of cardiovascular disease. Blood pressure measured after five minutes of seated rest; the mean of two consecutive measurements was used for analysis.
Anthropometric assessment	Height and weight measured with participants wearing light clothing and no shoes. Body mass index (BMI) calculated as weight (kg)/height^2^ (m^2^). Waist circumference measured at the midpoint between the lower rib margin and the iliac crest.
Laboratory evaluation	Overnight fasting venous blood samples collected for fasting plasma glucose, glycated haemoglobin (HbA1c), serum creatinine, and total cholesterol, low-density lipoprotein cholesterol (LDL-C), high-density lipoprotein cholesterol (HDL-C), and triglycerides.

### 2.2 Clinical assessment and cardiovascular risk factors definitions

A standardized clinical assessment was performed for all participants. Demographic characteristics, smoking status, medical history, medication use, and family history of cardiovascular disease were recorded. Height and weight were measured with participants wearing light clothing and no shoes. Body mass index (BMI) was calculated as weight (kg)/height^2^ (m^2^), and waist circumference was measured at the midpoint between the lower rib margin and the iliac crest using a non-stretch measuring tape. Blood pressure was measured after five minutes of seated rest using a calibrated sphygmomanometer, and the mean of two consecutive measurements was used for analysis.

Following an overnight fast, venous blood samples were collected for fasting plasma glucose, glycated haemoglobin (HbA1c), serum creatinine, and a fasting lipid profile. Cardiovascular risk factors were defined using the INTERHEART study framework (6) with hypertension classified according to the 2017 American College of Cardiology/American Heart Association (ACC/AHA) guideline (28). Operational definitions of cardiovascular risk factors are presented in Table 2.

**Table 2:** Definitions of cardiovascular risk factors.

Risk factor	Definition
Hypertension	Previous diagnosis of hypertension, current antihypertensive therapy, or blood pressure ≥130/80 mmHg, consistent with the 2017 ACC/AHA guideline (28)
Diabetes mellitus	Previous diagnosis of diabetes mellitus, current glucose-lowering therapy, fasting plasma glucose ≥126 mg/dL (7.0 mmol/L), or glycated haemoglobin (HbA1c ≥ 6.5%).
Dyslipidaemia	Previous diagnosis of dyslipidaemia, current lipid-lowering therapy, or abnormal fasting lipid profile, including total cholesterol >200 mg/dL, triglycerides >150 mg/dL, low-density lipoprotein cholesterol (LDL-C > 100 mg/dL), or high-density lipoprotein cholesterol (HDL-C < 40 mg/dL in men or <50 mg/dL in women).
Obesity	Body mass index (BMI) ≥ 30 kg/m².
Smoking	Current tobacco use.
Family history	Coronary artery disease (CAD), chronic kidney disease (CKD), or cardiovascular disease (CVD) in a first-degree relative.
Physical inactivity	As defined according to the INTERHEART study framework (6)
Psychological problems	As defined according to the INTERHEART study framework (6)

### 2.3 Echocardiographic assessment of epicardial fat thickness and carotid ultrasonographic assessment of intima-media thickness

All participants underwent transthoracic echocardiography using a Vivid E95 ultrasound system (GE Healthcare, Chicago, IL, USA). Standard echocardiographic views were acquired according to the recommendations of the American Society of Echocardiography. Standard two-dimensional, M-mode, pulsed-wave Doppler, and tissue Doppler imaging were performed to assess left ventricular systolic and diastolic function and left ventricular mass. Left ventricular mass was calculated using the Devereux and Reichek cube formula and indexed to body surface area to derive the left ventricular mass index (LVMI).

Epicardial adipose tissue (EAT) is the visceral fat depot located between the myocardium and the visceral pericardium. Epicardial fat thickness (EFT) represents a linear echocardiographic measure of this adipose tissue and was used as a surrogate marker of epicardial adiposity.

Epicardial fat thickness (EFT) was measured from the transthoracic echocardiographic examination with participants in the left lateral decubitus position. In the parasternal long-axis view, EFT was identified as the echo-free space between the outer wall of the myocardium and the visceral pericardium overlying the free wall of the right ventricle. Measurements were obtained perpendicular to the right ventricular free wall at end-systole, using the aortic annulus as an anatomical landmark, according to the standardized echocardiographic methodology originally described by Iacobellis and colleagues and adopted in subsequent studies (29, 30). Three consecutive cardiac cycles were measured, and the mean value was used for analysis to improve measurement reproducibility. End-systolic measurements were selected because the epicardial fat layer is more clearly delineated and less susceptible to compression during the cardiac cycle, improving measurement reliability (29, 30).

**Figure 1:**
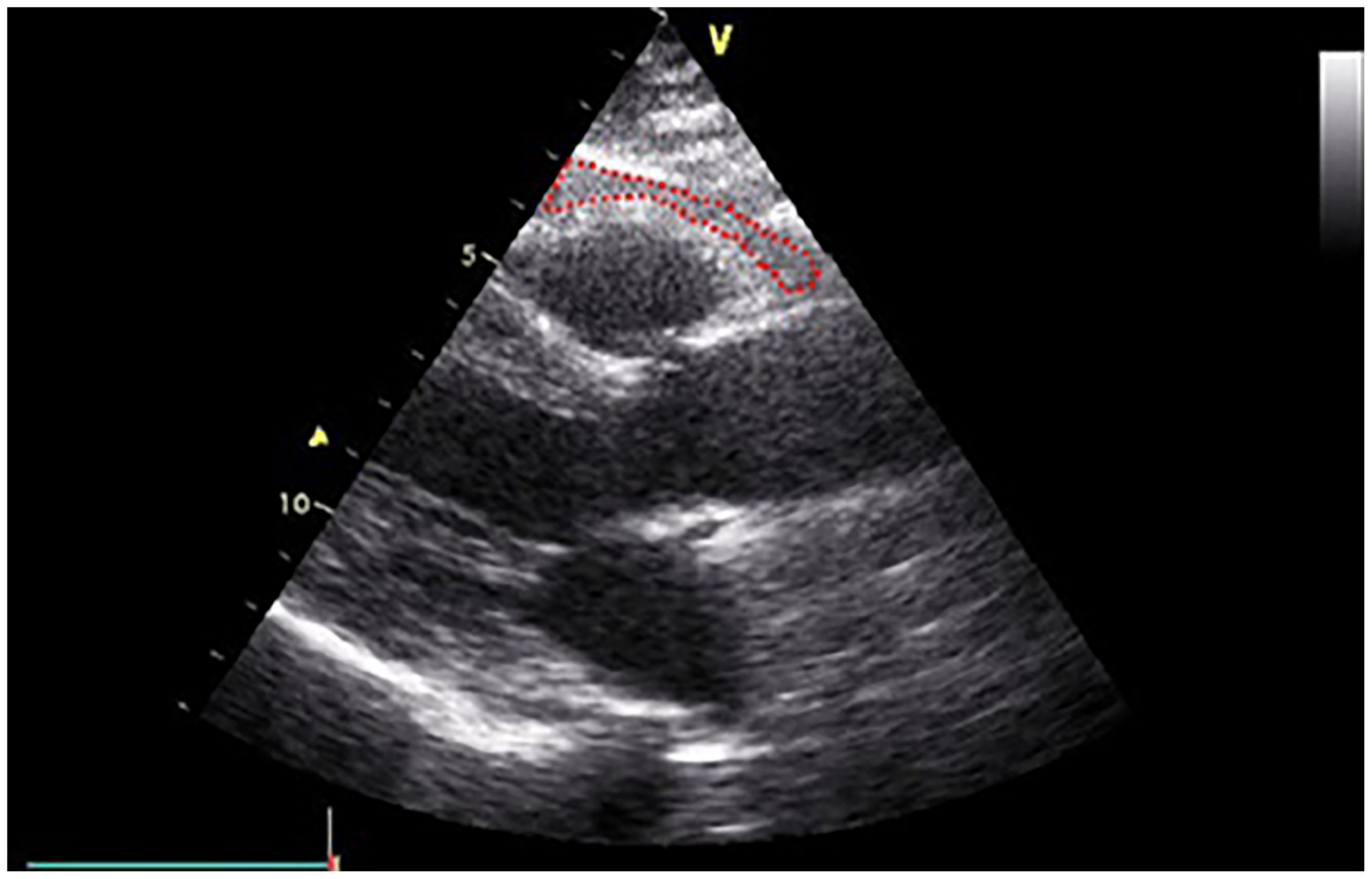
Echocardiographic measurement of epicardial fat thickness (EFT). Epicardial fat thickness was measured in the parasternal long-axis view as the echo-free space between the outer wall of the myocardium and the visceral pericardium overlying the free wall of the right ventricle. Measurements were obtained at end-systole, perpendicular to the right ventricular free wall, using the aortic annulus as an anatomical landmark.

Previous studies have demonstrated that increasing epicardial fat thickness is associated with the presence and severity of coronary artery disease; however, reported echocardiographic cut-off values vary across studies, and no universally accepted echocardiographic threshold has been established for cardiovascular risk assessment in asymptomatic populations (30, 31). Therefore, EFT was analysed as a continuous variable throughout the study (Figure 1).

**Figure 2:**
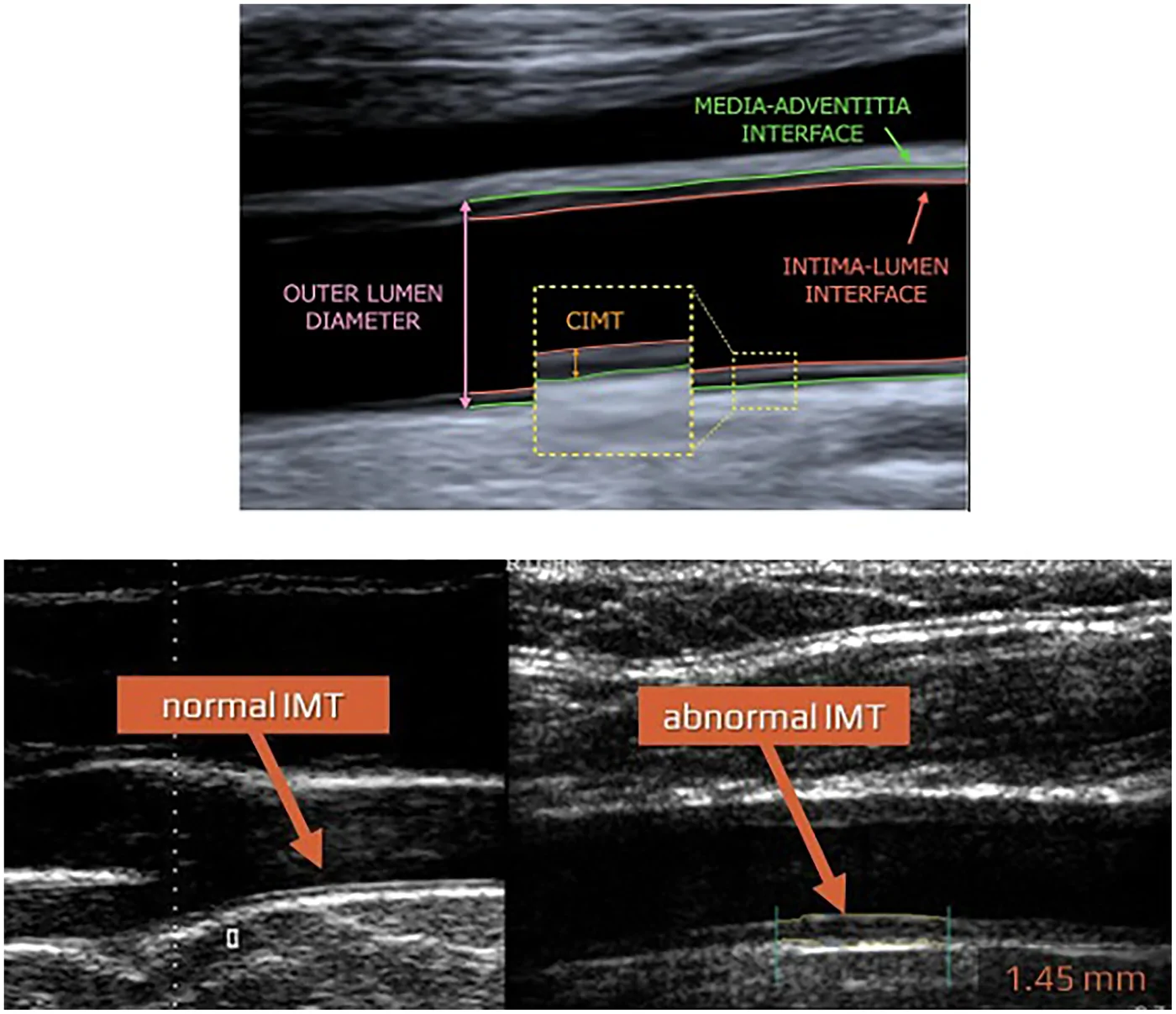
Measurement of carotid intima-media thickness (cIMT). High-resolution B-mode ultrasonography demonstrating measurement of the far wall of the distal common carotid artery approximately 1 cm proximal to the carotid bulb. Three measurements were obtained from each carotid artery and averaged for analysis.

Carotid intima-media thickness (cIMT) was measured using a 10–14 MHz linear-array transducer with participants in the supine position and the neck slightly extended. High-resolution B-mode imaging with ECG gating was used to obtain measurements from the far wall of the distal 1 cm of the common carotid artery proximal to the carotid bulb on both the left and right sides. Three measurements were obtained from each carotid artery, and the mean value was used for analysis. Carotid imaging was performed according to the recommendations of the American Society of Echocardiography (Figure 2) (11, 16).

For descriptive analyses cIMT values were categorised as normal (≤0.8 mm), borderline (0.9 mm), and abnormal (≥1.0 mm) according to the predefined study protocol. When examining the prevalence of abnormal cIMT across age groups, participants with borderline cIMT (0.9 mm) were grouped with the normal category, and abnormal cIMT was defined as ≥1.0 mm. Continuous cIMT measurements were retained for all primary statistical analyses. Focal wall thickening ≥1.5 mm was classified as carotid plaque in accordance with the Mannheim carotid intima-media thickness Consensus.

#### 2.3.1 Quality assurance

Standardized imaging protocols were used throughout the study. All echocardiographic and carotid ultrasound examinations were performed by experienced operators using standardized acquisition protocols. Image analysis was performed by observers blinded to participants' clinical characteristics and cardiovascular risk-factor status. Standardized measurement procedures were used throughout the study to enhance measurement consistency and reproducibility. Routine quality assurance procedures, including standardized operator training and adherence to predefined imaging protocols, were implemented throughout the study to minimise inter-observer variability.

### 2.4 Statistical analysis

Statistical analyses were performed using Python version 3.14 (Python Software Foundation, Wilmington, DE, USA) using the SciPy library scientific computing library version 1.17.1 (32). Continuous variables are presented as mean ± standard deviation (SD) while categorical variables are presented as frequencies and percentages.

Comparisons between two groups were performed using the independent-samples *t*-test for continuous variables and the chi-square test for categorical variables. Comparisons across multiple groups were performed using one-way analysis of variance (ANOVA). Left and right cIMT measurements were compared using paired-samples *t*-tests. A two-sided *p*-value of <0.05 was considered statistically significant.

### 2.5 Ethical approval

This study was approved by the Institutional Ethics Committee of Sri Ramachandra Institute of Higher Education and Research, Chennai, India (Approval No. CSP/23/DEC/141/966). Written informed consent was obtained from all participants prior to enrolment. All study procedures were conducted in accordance with the ethical principles of the Declaration of Helsinki and its subsequent amendments.

## 3 Results

### 3.1 Participant characteristics

A total of 925 asymptomatic adults were included in the study, comprising 179 participants without cardiovascular risk factors (control group) and 746 participants with one or more cardiovascular risk factors.

Participants with cardiovascular risk factors were significantly older than controls (52.3 ± 11.5 vs. 44.8 ± 9.6 years, *p* < 0.001). They also had a significantly greater waist circumference (94.7 ± 12.1 vs. 90.4 ± 12.2 cm, *p* < 0.001), higher HbA1c levels (6.9 ± 1.7% vs. 6.0 ± 1.0%, *p* < 0.001), higher triglyceride concentrations (152.3 ± 85.9 vs. 134.7 ± 90.5 mg/dL, *p* = 0.015), and a modestly higher body mass index (28.0 ± 4.7 vs. 27.3 ± 4.5 kg/m^2^, *p* = 0.049).

No statistically significant differences were observed between the groups for serum creatinine (0.9 ± 0.5 vs. 0.8 ± 0.2 mg/dL, *p* = 0.271), HDL cholesterol (44.8 ± 9.2 vs. 45.1 ± 8.6 mg/dL, *p* = 0.661), LDL cholesterol (133.1 ± 34.6 vs. 129.0 ± 31.2 mg/dL, *p* = 0.148), or total cholesterol (197.0 ± 43.5 vs. 193.7 ± 42.5 mg/dL, *p* = 0.361). Baseline demographic and clinical characteristics are summarised in Table 3.

**Table 3:** Baseline demographic and clinical characteristics of the study population according to cardiovascular risk-factor status.

Characteristic	Control (*n* = 179)	≥1 Cardiovascular Risk Factor (*n* = 746)	*p*-value
Age (years)	44.8 ± 9.6	52.3 ± 11.5	<0.001
Waist circumference (cm)	90.4 ± 12.2	94.7 ± 12.1	<0.001
HbA1c (%)^a^	6.0 ± 1.0	6.9 ± 1.7	<0.001
Serum creatinine (mg/dL)^b^	0.8 ± 0.2	0.9 ± 0.5	0.271
HDL cholesterol (mg/dL)	45.1 ± 8.6	44.8 ± 9.2	0.661
LDL cholesterol (mg/dL)	129.0 ± 31.2	133.1 ± 34.6	0.148
Triglycerides (mg/dL)	134.7 ± 90.5	152.3 ± 85.9	0.015
Total cholesterol (mg/dL)	193.7 ± 42.5	197.0 ± 43.5	0.361
Body mass index (kg/m^2^)	27.3 ± 4.5	28.0 ± 4.7	0.049

Values are presented as mean ± standard deviation (SD). Comparisons between groups were performed using the independent-samples *t*-test.

a HbA1c measurements were unavailable for 80 participants and were excluded from the corresponding analysis.

b Serum creatinine measurements were unavailable for 46 participants and were excluded from the corresponding analysis.

### 3.2 Echocardiographic and carotid ultrasonographic findings

Echocardiographic and carotid ultrasonographic measurements are summarised in Table 4. Participants with one or more cardiovascular risk factors demonstrated greater carotid intima-media thickness (cIMT) and epicardial fat thickness (EFT) than those without cardiovascular risk factors.

**Table 4:** Echocardiographic and carotid ultrasonographic measurements according to cardiovascular risk-factor status.

Variable	Control (*n* = 179)	≥1 Cardiovascular Risk Factor (*n* = 746)	*P* value
Right cIMT (mm)	0.576 ± 0.144	0.681 ± 0.186	<0.001
Left cIMT (mm)	0.587 ± 0.143	0.711 **±** 0.193	<0.001
Epicardial fat thickness (mm)	4.24 ± 1.85	5.45 ± 2.30	<0.001

Values are presented as mean ± standard deviation (SD). Comparisons between groups were performed using the independent-samples *t*-test. cIMT, carotid intima-media thickness; EFT, epicardial fat thickness.

Mean right cIMT was higher in participants with cardiovascular risk factors than in controls (0.681 ± 0.186 mm vs. 0.576 ± 0.144 mm, *p* < 0.001). Similarly, mean left cIMT was greater in the cardiovascular risk group (0.711 ± 0.193 mm vs. 0.587 ± 0.143 mm, *p* < 0.001). Mean EFT was also higher among participants with cardiovascular risk factors (5.45 ± 2.30 mm vs. 4.24 ± 1.85 mm, *p* < 0.001).

Across the entire study population (*n* = 925), mean left cIMT was significantly greater than mean right cIMT (0.687 ± 0.190 mm vs. 0.661 ± 0.182 mm, respectively). The mean paired difference was 0.026 mm (95% CI 0.016–0.036 mm), which was statistically significant (paired *t*-test: *t* = 4.95, *p* < 0.001).

### 3.3 Distribution of carotid intima-media thickness categories

The prevalence of abnormal cIMT increased significantly with advancing age (Table 5). Abnormal cIMT was identified in 2.2% (4/179) of participants younger than 40 years, increasing to 8.5% (46/539) among those aged 40–60 years and 28.0% (58/207) among participants older than 60 years. Overall, abnormal cIMT was present in 108 of 925 participants (11.7%). There was a significant association between advancing age and abnormal cIMT (*χ*^2^ = 74.25, df = 2, *p* < 0.001).

**Table 5:** Association between Age group and abnormal cIMT.

Age Group (years)	Normal cIMT *n* (%)	Abnormal cIMT *n* (%)	Total, *n*
<40	175 (97.8%)	4 (2.2%)	179
40–60	493 (91.5%)	46 (8.5%)	539
>60	149 (72.0%)	58 (28.0%)	207
Total	817 (88.3%)	108 **(**11.7%)	925

Data are presented as number (%). Pearson's chi-square test was used to compare the distribution of abnormal cIMT across age groups. cIMT, carotid intima-media thickness. *χ*^2^ = 74.25, df = 2, *p* < 0.001.

### 3.4 Association between cardiovascular risk factors and abnormal carotid intima–media thickness

Hypertension, diabetes mellitus, dyslipidaemia, and chronic kidney disease were significantly more prevalent among participants with abnormal cIMT than those with normal cIMT (all *p* < 0.05). In contrast, family history, smoking, psychological stress, and physical inactivity were not significantly associated with abnormal cIMT (Table 6).

**Table 6:** Cardiovascular risk factors associated with abnormal cIMT.

Risk factor	Normal cIMT (*n* = 817)	Abnormal cIMT (*n* = 108)	*p*-value
Hypertension	251 (30.7%)	59 (54.6%)	<0.001
Diabetes mellitus	225 (27.5%)	51 (47.2%)	<0.001
Dyslipidaemia	115 (14.1%)	28 (25.9%)	0.001
Family history	158 (19.3%)	22 (20.4%)	0.799
Smoking	99 (12.1%)	13 (12.0%)	0.979
Psychological stress	159 (19.5%)	20 (18.5%)	0.428
Physical inactivity	207 (25.3%)	28 (25.9%)	0.895
Chronic kidney disease	37 (4.5%)	11 (10.2%)	0.024

Values are presented as number (%). Comparisons between participants with normal and abnormal carotid intima-media thickness (cIMT) were performed using Pearson's chi-square test. cIMT, carotid intima-media thickness; CKD, chronic kidney disease.

### 3.5 Association between individual cardiovascular risk factors and imaging findings

Associations between individual cardiovascular risk factors and imaging measurements are summarised in Table 7. Hypertension, diabetes mellitus, and dyslipidaemia were each associated with significantly greater mean carotid intima-media thickness (cIMT) and epicardial fat thickness (EFT) than in participants without these conditions (all *p* < 0.001). Chronic kidney disease was associated with significantly greater mean EFT (*p* = 0.028) but was not significantly associated with cIMT (*p* = 0.099).

**Table 7:** Association between individual cardiovascular risk factors and imaging findings.

Cardiovascular Risk Factor	n	Mean cIMT (mm)	*p*-value	Mean EFT (mm)	*p*-value
Hypertension					
Has HTN	310	0.732	<0.001	6.057	<0.001
No HTN	615	0.645		4.893	
Diabetes mellitus					
Has DM	276	0.725	<0.001	5.832	<0.001
No DM	649	0.652		5.050	
Dyslipidaemia					
Has DLP	143	0.734	<0.001	6.021	<0.001
No DLP	782	0.663		5.148	
Family history of cardiovascular disease					
Has FH	180	0.672	0.855	5.383	0.940
No FH	745	0.674		5.259	
Current smoking					
Current smoker	112	0.671	0.840	5.144	0.255
Non-smoker	813	0.674		5.302	
Chronic kidney disease					
Has CKD	48	0.713	0.099	5.963	0.028
No CKD	877	0.672		5.246	

Values are presented as mean cIMT and mean EFT. Comparisons between participants with and without each cardiovascular risk factor were performed using independent-samples *t*-tests. cIMT, carotid intima-media thickness; EFT, epicardial fat thickness; HTN, hypertension; DM, diabetes mellitus; DLP, dyslipidaemia; FH, family history of cardiovascular disease; CKD, chronic kidney disease.

In contrast, family history of cardiovascular disease and current smoking were not significantly associated with either cIMT or EFT (all *p* > 0.05).

### 3.6 Association between cumulative cardiovascular risk-factor burden and imaging findings

Increasing cardiovascular risk-factor burden was associated with progressive increases in both carotid intima-media thickness (cIMT) and epicardial fat thickness (EFT) (Table 8). Controls had the lowest mean cIMT (0.58 mm) and mean EFT (4.24 mm), whereas those with three or more cardiovascular risk factors demonstrated the highest mean cIMT (0.71 mm) and mean EFT (6.05 mm). One-way analysis of variance demonstrated significant differences across the four groups for both cIMT (F = 24.8, *p* < 0.001) and EFT (F = 22.8, *p* < 0.001).

**Table 8:** Mean carotid intima–media thickness and epicardial fat thickness according to cardiovascular risk-factor burden.

Number of cardiovascular risk factors	*n*	Mean cIMT (mm)	Mean EFT (mm)
0	179	0.58	4.24
1	332	0.69	5.26
2	239	0.69	5.53
≥3	175	0.71	6.05
Overall ANOVA (F, *p*-value)		F = 24.8, *p* < 0.001	*F* = 22.8, *p* < 0.001

Values are presented as group means. Comparisons across groups were performed using one-way analysis of variance (ANOVA). cIMT, carotid intima-media thickness; EFT, epicardial fat thickness.

## 4 Discussion

Cardiovascular disease remains the leading cause of morbidity and mortality worldwide, with South Asian populations experiencing an earlier onset of disease and a disproportionately high burden of traditional cardiovascular risk factors (1, 2, 5–8). In this cross-sectional study of 925 asymptomatic adults undergoing routine cardiovascular health assessment, we evaluated the associations between carotid intima-media thickness (cIMT), epicardial fat thickness (EFT), and traditional cardiovascular risk factors. Participants with one or more cardiovascular risk factors demonstrated significantly greater cIMT and EFT than controls. Furthermore, advancing age and increasing cardiovascular risk-factor burden were associated with progressive increases in both imaging markers. Collectively, these findings support the role of cIMT and EFT as complementary non-invasive imaging markers of subclinical vascular remodelling and cardiovascular risk.

### 4.1 Carotid intima-media thickness and cardiovascular risk factors

Carotid intima-media thickness is a well-established surrogate marker of vascular remodelling and cardiovascular risk that reflects structural changes within the arterial wall (11–16, 26). In the present study, participants with hypertension, diabetes mellitus, and dyslipidaemia demonstrated significantly greater cIMT than those without these risk factors. These findings are consistent with previous studies demonstrating that traditional cardiovascular risk factors accelerate vascular ageing and arterial wall thickening (11–16, 26).

Hypertension demonstrated the strongest association with increased cIMT, followed closely by diabetes mellitus and dyslipidaemia. These observations are biologically plausible, as chronic hypertension promotes medial hypertrophy and endothelial dysfunction, diabetes accelerates glycation and vascular inflammation, and dyslipidaemia contributes to lipid deposition and arterial remodelling (4, 11–16, 26). Together, these findings reinforce the importance of comprehensive cardiovascular risk-factor control to reduce the burden of progressive vascular remodelling.

### 4.2 Epicardial fat thickness

EFT is increasingly recognised as a marker of metabolically active visceral adiposity and systemic inflammation, reflecting the close interaction between adipose tissue, vascular dysfunction, and atherosclerosis (17–23). In our cohort, participants with hypertension, diabetes mellitus, and dyslipidaemia demonstrated significantly greater EFT, supporting previous reports linking epicardial adipose tissue with adverse cardiometabolic profiles (17–23). Recent studies have further strengthened the evidence for EFT as a clinically relevant imaging marker. Charisopoulou et al. (17) demonstrated that EFT measured by echocardiography was independently associated with coronary artery disease severity and myocardial ischaemia, with participants exhibiting EFT > 5 mm having significantly greater angiographic disease burden. Similarly, Bouzidi et al. (18) reported that increased EFT was independently associated with obstructive coronary artery disease and correlated with both Gensini and SYNTAX scores, highlighting its potential value as a non-invasive marker of cardiovascular risk.

Although both cIMT and EFT were significantly associated with traditional cardiovascular risk factors, cIMT demonstrated more consistent associations across the study population. In contrast, neither smoking nor family history was significantly associated with either imaging marker, suggesting that these variables contributed less to subclinical vascular remodelling within this asymptomatic cohort. Unlike the studies by Charisopoulou et al. (17) and Bouzidi et al. (18) which evaluated patients undergoing investigation for suspected coronary artery disease, our study examined an asymptomatic screening population without direct coronary imaging. Nevertheless, despite differences in study populations and study design, our findings support the potential role of EFT as a complementary marker of cardiometabolic risk in asymptomatic adults rather than a diagnostic marker of obstructive coronary artery disease.

### 4.3 Age and cardiovascular risk-factor burden

One of the principal findings of this study was the strong relationship between age and abnormal cIMT. The prevalence of abnormal cIMT increased progressively from 2.2% in participants younger than 40 years to 28.0% in those older than 60 years. This finding is consistent with the established concept of vascular ageing, whereby cumulative exposure to cardiovascular risk factors results in progressive arterial wall remodelling over time (11–15, 26).

Similarly, increasing cardiovascular risk-factor burden was associated with progressive increases in both cIMT and EFT. Participants with no cardiovascular risk factors had the lowest imaging measurements, whereas those with three or more risk factors demonstrated the greatest arterial wall thickness and epicardial fat accumulation. These findings suggest that the cumulative burden of cardiovascular risk factors is associated with greater vascular remodelling than individual risk factors considered in isolation and support the concept of integrated cardiovascular risk assessment (9–12, 17–19, 23).

### 4.4 Left–right differences in carotid intima-media thickness

We observed a modest but statistically significant difference between left and right carotid intima-media thickness (cIMT), with greater mean values on the left (mean paired difference 0.026 mm, 95% CI 0.016–0.036 mm). This finding is consistent with previous studies demonstrating greater cIMT in the left than the right common carotid artery, including the population-based study by Stevens et al. (33), and earlier work by Luo et al. (34). Anatomically, the left common carotid artery arises directly from the aortic arch, whereas the right originates from the brachiocephalic trunk, resulting in differences in haemodynamic forces and shear stress that may contribute to asymmetric vascular remodelling (11, 33–35). Although the observed left–right difference was small, it highlights the importance of bilateral carotid assessment during ultrasound examination. However, the clinical significance of this laterality remains uncertain, and further prospective longitudinal studies are needed to determine whether side-specific differences in cIMT have incremental prognostic value for cardiovascular risk prediction (16, 33, 35).

### 4.5 Clinical implications

Our findings support the use of carotid intima-media thickness (cIMT) and epicardial fat thickness (EFT) as complementary non-invasive imaging markers associated with cardiovascular risk in asymptomatic adults. In the present study, cIMT demonstrated more consistent associations with traditional cardiovascular risk factors and increasing cardiovascular risk-factor burden than EFT, whereas EFT appeared to provide complementary information regarding metabolic cardiovascular risk. These complementary characteristics suggest that combined assessment of cIMT and EFT may enhance conventional cardiovascular risk assessment, particularly in South Asian populations, where cardiovascular disease develops at a younger age and conventional cardiovascular risk prediction models may not fully capture lifetime cardiovascular risk (3, 5–10, 27, 36).

The observed association between dyslipidaemia and increased cIMT may also have therapeutic implications. Although this cross-sectional study cannot determine whether reducing cIMT improves clinical outcomes, identification of individuals with increased cIMT may support earlier implementation of lifestyle modification and guideline-directed lipid-lowering therapy in individuals at elevated cardiovascular risk. This approach is consistent with the 2021 ESC Guidelines on cardiovascular disease prevention in clinical practice, the 2025 ESC/EAS Focused Update on the management of dyslipidaemias, and expert recommendations for cardiovascular risk assessment in South Asian populations (9, 10, 37). However, future prospective longitudinal studies are needed to determine whether incorporating cIMT and EFT into routine cardiovascular risk assessment improves clinical outcomes.

### 4.6 Limitations

This study has several limitations. First, the cross-sectional design precludes conclusions regarding causality or the temporal sequence of the observed associations between cardiovascular risk factors and vascular imaging markers. In addition, the absence of longitudinal follow-up prevented assessment of whether participants with increased carotid intima-media thickness (cIMT) or epicardial fat thickness (EFT) subsequently developed major adverse cardiovascular events, including myocardial infarction, stroke, or cardiovascular mortality.

Second, the study population comprised asymptomatic adults attending routine cardiovascular health assessments. Although this reflects real-world clinical practice, the proportion of participants with one or more cardiovascular risk factors was substantially greater than that of the control group, which may have influenced the precision of between-group comparisons. Furthermore, the findings may not be generalisable to the broader population or to individuals with established cardiovascular disease.

Third, blood pressure was measured only in the left arm. Current European Society of Cardiology (ESC) and American College of Cardiology/American Heart Association (ACC/AHA) guidelines recommend measuring blood pressure in both arms during the initial assessment, with subsequent measurements taken from the arm with the higher reading for diagnosis and management. Consequently, some participants may have been misclassified with respect to hypertension status (36, 38).

Fourth, although cIMT and EFT are well-established non-invasive imaging markers associated with cardiovascular risk (12, 15, 26, 39), they represent surrogate markers of vascular remodelling rather than direct measures of coronary atherosclerosis. Direct assessment of coronary artery atherosclerosis using coronary CT angiography, coronary artery calcium scoring, invasive coronary angiography, or functional ischaemia testing was not performed. Accordingly, the findings should be interpreted as demonstrating associations between cardiovascular risk factors and surrogate imaging markers rather than direct evidence of coronary atherosclerosis.

Fifth, this was a single-centre study conducted in a South Indian population, which may limit the external validity of the findings.

Finally, formal intra- and inter-observer reproducibility analyses were not performed because repeat imaging measurements by independent observers were not prospectively collected. Although standardized imaging protocols were used throughout the study to minimise measurement variability, residual observer-related variation cannot be excluded. Future multicentre, prospective longitudinal studies involving more diverse populations, incorporating reproducibility analyses, direct coronary imaging where appropriate, and long-term clinical cardiovascular outcomes, are required to further establish the clinical utility of cIMT and EFT for cardiovascular risk stratification.

## 5 Conclusion

This study demonstrates that carotid intima-media thickness is consistently associated with traditional cardiovascular risk factors and increasing cardiovascular risk-factor burden in asymptomatic adults. Epicardial fat thickness was also associated with hypertension, diabetes mellitus and dyslipidaemia, suggesting that it may provide complementary information during cardiovascular risk stratification. Together, these complementary imaging markers may enhance cardiovascular risk stratification beyond conventional risk-factor assessment. These findings highlight the potential value of incorporating cIMT and EFT into non-invasive cardiovascular risk assessment, although prospective longitudinal studies are needed to determine their incremental predictive value for future cardiovascular events.

## Data Availability

The original contributions presented in the study are included in the article; further inquiries can be directed to the corresponding author.

## References

[1] Stark BA DeCleene NK Desai EC Hsu JM Johnson CO Lara-Castor L. Global, regional, and national burden of cardiovascular diseases and risk factors in 204 countries and territories, 1990–2023. J Am Coll Cardiol. (2025) 86(22):2167–243. 10.1016/j.jacc.2025.08.015 40990886

[2] World Health Organization. Cardiovascular Diseases (CVDs). Geneva: World Health Organization (2025). Available online at: WHO Cardiovascular diseases (CVDs) fact sheet(Accessed June 24, 2026).

[3] Gupta K Modi S Ananthasubramaniam K. Toward understanding cardiovascular risk burden in South Asians: a Major step forward. JACC: Asia. (2022) 2:912–5. 10.1016/j.jacasi.2022.10.005 36713758 PMC9877210

[4] Libby P. The changing landscape of atherosclerosis. Nature. (2021) 592(7855):524–33. 10.1038/s41586-021-03392-8 33883728

[5] Patel AP Wang M Kartoun U Ng K Khera AV. Quantifying and understanding the higher risk of atherosclerotic cardiovascular disease among South Asian individuals: results from the UK biobank prospective cohort study. Circulation. (2021) 144(6):410–22. 10.1161/CIRCULATIONAHA.120.052430 34247495 PMC8355171

[6] Yusuf S Hawken S Ôunpuu S Dans T Avezum A Lanas F. Effect of potentially modifiable risk factors associated with myocardial infarction in 52 countries (the INTERHEART study): case-control study. Lancet. (2004) 364(9438):937–52. 10.1016/S0140-6736(04)17018-9 15364185

[7] Pedamallu H Aghabazaz Z Lancki N Rodriguez LA Siddique J Moorthy M. Prevalence and trends in cardiovascular risk factors among middle-aged south Asian adults compared with other racial and ethnic groups in the United States: a longitudinal analysis of 2 cohort studies. J Am Heart Assoc. (2026) 15(4):1–13. 10.1161/JAHA.124.041221 PMC13055708 41669952

[8] Volgman AS Palaniappan LS Aggarwal NT Gupta M Khandelwal A Krishnan AV. Atherosclerotic cardiovascular disease in South Asians in the United States: epidemiology, risk factors, and treatments: a scientific statement from the American Heart Association. Circulation. (2018) 138(1):e1–34. 10.1161/CIR.0000000000000580 29794080

[9] Visseren FLJ Mach F Smulders YM Carballo D Koskinas KC Bäck M. 2021 ESC guidelines on cardiovascular disease prevention in clinical practice: developed by the task force for cardiovascular disease prevention in clinical practice with representatives of the European Society of Cardiology and 12 medical societies with the special contribution of the European association of preventive cardiology (EAPC). Eur Heart J. (2021) 42(34):3227–337. 10.1093/eurheartj/ehab484 34458905

[10] Agarwala A Satish P Al Rifai M Mehta A Cainzos-Achirica M Shah NS. Identification and management of atherosclerotic cardiovascular disease risk in South Asian populations in the U.S. JACC: Advances. (2023) 2(2):100258. 10.1016/j.jacadv.2023.100258 38089916 PMC10715803

[11] Stein JH Korcarz CE Hurst RT Lonn E Kendall CB Mohler ER. Use of carotid ultrasound to identify subclinical vascular disease and evaluate cardiovascular disease risk: a consensus statement from the American society of echocardiography carotid intima-media thickness task force endorsed by the society for vascular medicine. J Am Soc Echocardiogr. (2008) 21(2):93–111. 10.1016/j.echo.2007.11.011 18261694

[12] Lorenz MW Markus HS Bots ML Rosvall M Sitzer M. Prediction of clinical cardiovascular events with carotid intima-media thickness: a systematic review and meta-analysis. Circulation. (2007) 115(4):459–67. 10.1161/CIRCULATIONAHA.106.628875 17242284

[13] O’Leary DH Polak JF Kronmal RA Manolio TA Burke GL Wolfson SK. Carotid-artery intima and media thickness as a risk factor for myocardial infarction and stroke in older adults. Cardiovascular health study collaborative research group. N Engl J Med. (1999) 340(1):14–22. 10.1056/NEJM199901073400103 9878640

[14] Chambless LE Heiss G Folsom AR Rosamond W Szklo M Sharrett AR. Association of coronary heart disease incidence with carotid arterial wall thickness and major risk factors: the atherosclerosis risk in communities (ARIC) study, 1987–1993. Am J Epidemiol. (1997) 146(6):483–94. 10.1093/oxfordjournals.aje.a009302 9290509

[15] Bots ML Hoes AW Koudstaal PJ Hofman A Grobbee DE. Common carotid intima-media thickness and risk of stroke and myocardial infarction: the Rotterdam study. Circulation. (1997) 96(5):1432–7. 10.1161/01.CIR.96.5.1432 9315528

[16] Johri AM Nambi V Naqvi TZ Feinstein SB Kim ESH Park MM. Recommendations for the assessment of carotid arterial plaque by ultrasound for the characterization of atherosclerosis and evaluation of cardiovascular risk: from the American society of echocardiography. J Am Soc Echocardiogr. (2020) 33(8):917–33. 10.1016/j.echo.2020.04.021 32600741

[17] Charisopoulou D Iliopoulou S Koulaouzidis G Antoniou N Tsantekidis K Mavrogianni AD. Epicardial fat thickness as a marker of coronary artery disease severity and ischemic burden: a prospective echocardiographic study. J Clin Med. (2026) 15(2):657. 10.3390/jcm15020657 41598597 PMC12842374

[18] Bouzidi H Lamine H Zairi I Ayadi A Tlili G Mzoughi K. Epicardial fat thickness by transthoracic echocardiography as a predictor of obstructive coronary artery disease. J Clin Lipidol. (2026) 20(2):368–77. 10.1016/j.jacl.2025.10.070 41455636

[19] Isomitdinov B Yakhshimurodov U Soe Thet M Whiteford J Oo Y Oo AY. The association of epicardial adipose tissue measurement and severity of coronary artery disease: a systematic review and meta-analysis. Eur J Prev Cardiol. (2025) 32(Supplement_1):zwaf236.066. 10.1093/eurjpc/zwaf236.066

[20] Şengül C Özveren O. Epicardial adipose tissue: a review of physiology, pathophysiology, and clinical applications. The Anatol J Cardiol. (2013) 13(3):261. 10.5152/akd.2013.075 23395709

[21] Jeong J-W Jeong MH Yun KH Oh SK Park EM Kim YK. Echocardiographic epicardial fat thickness and coronary artery disease. Circ J. (2007) 71(4):536–9. 10.1253/circj.71.536 17384455

[22] Ahn S-G Lim H-S Joe D-Y Kang S-J Choi B-J Choi S-Y. Relationship of epicardial adipose tissue by echocardiography to coronary artery disease. Heart. (2008) 94(3):e7. 10.1136/hrt.2007.118471 17923467

[23] Iacobellis G. Epicardial adipose tissue in contemporary cardiology. Nat Rev Cardiol. (2022) 19(9):593–606. 10.1038/s41569-022-00679-9 35296869 PMC8926097

[24] Kocaman SA Baysan O Çetin M Altuner TK Ocaklı EP Durakoğlugil ME. An increase in epicardial adipose tissue is strongly associated with carotid intima-media thickness and atherosclerotic plaque, but LDL only with the plaque. Anatol J Cardiol. (2017) 17(1):56. 10.14744/AnatolJCardiol.2016.6885 27564776 PMC5324864

[25] Cetin M Cakici M Polat M Suner A Zencir C Ardic I. Relation of epicardial fat thickness with carotid intima-media thickness in patients with type 2 diabetes Mellitus. Int J Endocrinol. (2013) 2013:1–6. 10.1155/2013/769175 PMC3665232 23762053

[26] Fernández-Alvarez V Linares-Sánchez M López F Rinaldo A Ferlito A. Role of arterial stiffness and carotid intima-media thickness on subclinical atherosclerosis and cardiovascular risk assessment. Cardiol Ther. (2025) 14(3):367–83. 10.1007/s40119-025-00428-2 40684005 PMC12379674

[27] Patel A Nicholls SJ Chew DP Lin AK Natarajan P Nelson AJ. Drivers of atherosclerotic cardiovascular disease in south asians: current insights and future directions. JACC: Asia. (2026) 6(8 Part 2):1627–45. 10.1016/j.jacasi.2025.10.024 41712894 PMC13491095

[28] Wang MC Petito LC Pool LR Foti K Juraschek SP McEvoy JW. The 2017 American College of Cardiology/American Heart Association hypertension guideline and blood pressure in older adults. Am J Prev Med. (2023) 65(4):640–8. 10.1016/j.amepre.2023.04.011 37105448 PMC10524146

[29] Iacobellis G Willens HJ Barbaro G Sharma AM. Threshold values of high-risk echocardiographic epicardial fat thickness. Obesity (Silver Spring). (2008) 16(4):887–92. 10.1038/oby.2008.6 18379565

[30] Iacobellis G Willens HJ. Echocardiographic epicardial fat: a review of research and clinical applications. J Am Soc Echocardiogr. (2009) 22(12):1311–9. 10.1016/j.echo.2009.10.013 19944955

[31] Eroglu S Sade LE Yildirir A Bal U Ozbicer S Ozgul AS. Epicardial adipose tissue thickness by echocardiography is a marker for the presence and severity of coronary artery disease. Nutr Metab Cardiovasc Dis. (2009) 19(3):211–7. 10.1016/j.numecd.2008.05.002 18718744

[32] Virtanen P Gommers R Oliphant TE Haberland M Reddy T Cournapeau D. Scipy 1.0: fundamental algorithms for scientific computing in python. Nat Methods. (2020) 17(3):261–72. 10.1038/s41592-019-0686-2 32015543 PMC7056644

[33] Stevens B Abdool-Carrim T Woodiwiss AJ. Left versus right carotid artery IMT: differential impact of age, gender, and cardiovascular risk factors. Int J Cardiovasc Imaging. (2024) 40(11):2391–404. 10.1007/s10554-024-03245-1 39325213 PMC11561018

[34] Luo X Yang Y Cao T Li Z. Differences in left and right carotid intima–media thickness and the associated risk factors. Clin Radiol. (2011) 66(5):393–8. 10.1016/j.crad.2010.12.002 21324442

[35] Saleh C. Left versus right common carotid artery intima-media thickness: when sub-millimetric differences matter. Int J Cardiovasc Imaging. (2024) 40(12):2631–2. 10.1007/s10554-024-03266-w 39466495

[36] McEvoy JW McCarthy CP Bruno RM Brouwers S Canavan MD Ceconi C. 2024 ESC guidelines for the management of elevated blood pressure and hypertension: developed by the task force on the management of elevated blood pressure and hypertension of the European Society of Cardiology (ESC) and endorsed by the European Society of Endocrinology (ESE) and the European stroke organisation (ESO). Eur Heart J. (2024) 45(38):3912–4018. 10.1093/eurheartj/ehae178 39210715

[37] Mach F Koskinas KC Roeters van Lennep JE Tokgözoğlu L Badimon L Baigent C. 2025 Focused update of the 2019 ESC/EAS guidelines for the management of dyslipidaemias: developed by the task force for the management of dyslipidaemias of the European Society of Cardiology (ESC) and the European atherosclerosis society (EAS). Eur Heart J. (2025) 46(42):4359–78. 10.1093/eurheartj/ehaf190 40878289

[38] Jones DW Ferdinand KC Taler SJ Johnson HM Shimbo D Abdalla M. 2025 AHA/ACC/AANP/AAPA/ABC/ACCP/ACPM/AGS/AMA/ASPC/NMA/PCNA/SGIM guideline for the prevention, detection, evaluation and management of high blood pressure in adults: a report of the American College of Cardiology/American Heart Association joint committee on clinical practice guidelines. Circulation. (2025) 152(11):e114–218. 10.1161/CIR.0000000000001356 40811497

[39] Xu Y Cheng X Hong K Huang C Wan L. How to interpret epicardial adipose tissue as a cause of coronary artery disease: a meta-analysis. Coron Artery Dis. (2012) 23(4):227–33. 10.1097/MCA.0b013e328351ab2c 22361934

